# Supplementary material for: Sustainable innovation in the context of organizational cultural diversity: The role of cultural intelligence and knowledge sharing
Source: PLoS One. 2021 May 19;16(5):e0250878. doi: 10.1371/journal.pone.0250878 (PMC8133532; doi:10.1371/journal.pone.0250878)
Supplement: S1 Appendix — (DOCX) [file pone.0250878.s001.docx]

CQ and Sustainable Innovation Behavior Questionnaire

Dear Sir/madam:

Hello! Thank you very much for participating in this survey! The purpose of this survey is to study problems at work. The survey will be conducted anonymously. The results of the questionnaire will only be used for academic research and will not involve ethical issues. All information about you will be kept confidential and approved by the Academic Ethics Committee of the University. Please answer the following questions according to your true feelings. There is no difference between good or bad, right or wrong. Please complete this form for valid results.

**1.Basic Information Section**

1.Gender：□ male □ female

2.Age：□20-25 □26-30 □31-35 □36-40 □41-45 □46 above

3. Education：□Technical secondary school □Junior college □Regular college

□Postgraduate or above

4. Work Years；□1 year □1-2 year □3-5 year □6-10 year □11 year above

5. Job rank：□ Grass-roots employee □ First-level manager □ Middle-manager

□ Top managers

6. Job category：□Marketing/Sales □R & D/Design □Engineering Support □Finance

□ General management □ Technology Services □Production/Operations □Other__

7. The nature of your organization：□State-owned enterprise □Privately □Foreign □Other__

8.Your company's industry belongs to：□Internet □E-Business □Finance □Manufacturing □Professional services/consulting □Real estate business □Other__

9.The size of your company is：□0-50 □51-100 □101-300 □301-1000 □1001above

**2.The questionnaire content**

***Cultural Intelligence（CQ）***

1.I am conscious of the cultural knowledge I use when interacting with people with different cultural backgrounds.

1□ 2□ 3□ 4 □5 □6 □7 □

2.I am conscious of the cultural knowledge I apply to cross-cultural interactions.

1□ 2□ 3□ 4 □5 □6 □7 □

3.I know the legal and economic systems of other cultures.

1□ 2□ 3□ 4 □5 □6 □7 □

4.I know the rules (e.g. vocabulary, grammar) of other languages.

1□ 2□ 3□ 4 □5 □6 □7 □

5.I know the cultural values and religious beliefs of other cultures.

1□ 2□ 3□ 4 □5 □6 □7 □

6.I know the marriage systems of other cultures.

1□ 2□ 3□ 4 □5 □6 □7 □

7.I know the arts and crafts of other cultures.

1□ 2□ 3□ 4 □5 □6 □7 □

8.I am confident that I can socialize with locals in a culture that is unfamiliar to me.

1□ 2□ 3□ 4 □5 □6 □7 □

9.I am sure I can deal with the stresses of adjusting to a culture that is new to me.

1□ 2□ 3□ 4 □5 □6 □7 □

10.I use pause and silence differently to suit different cross-cultural situations.

1□ 2□ 3□ 4 □5 □6 □7 □

11.I vary the rate of my speaking when a cross-cultural situation requires it.

1□ 2□ 3□ 4 □5 □6 □7 □

12.I change my nonverbal behavior when a cross-cultural interaction requires it.

1□ 2□ 3□ 4 □5 □6 □7 □

***Knowledge Sharing（KS）***

1.The members of my organization share their unique knowledge and expertise with each other.

1□ 2□ 3□ 4 □5 □6 □7 □

2.They may share knowledge or information when other members ask for special knowledge to accomplish team tasks.

1□ 2□ 3□ 4 □5 □6 □7 □

3.There is an exchange of knowledge and skills among members of my organization (company).

1□ 2□ 3□ 4 □5 □6 □7 □

4.The more knowledgeable members will provide the other members with knowledge or skills that are difficult to acquire for free.

1□ 2□ 3□ 4 □5 □6 □7 □

***Different organizational management styles***

1.The firm/partner relies on an informal organization (e.g., has few managerial layers, loose control and monitoring; would settle for a handshake instead of sticking to bureaucratic procedures, contracts and legal documentation)

1□ 2□ 3□ 4 □5 □6 □7 □

2.The firm/partner uses consensus seeking rather than authoritarian decision making (e.g., many people are democratically involved in decisions instead of one senior person making all the calls)

1□ 2□ 3□ 4 □5 □6 □7 □

3.The firm/partner prefers informal over formal communication (e.g., bullet-point presentations or verbal communication instead of lengthy written reports)

1□ 2□ 3□ 4 □5 □6 □7 □

4.The firm/partner has an apolitical organization (e.g., decisions are guided by concrete considerations and planned processes rather than by personalities and hidden agendas)

1□ 2□ 3□ 4 □5 □6 □7 □

***Organizational responsiveness styles***

1.The firm/partner trusts outsiders unless given a reason not to (e.g., willingly shares sensitive information, such as future product introductions, allowing other firms on customer sites)

1□ 2□ 3□ 4 □5 □6 □7 □

2.The firm/partner has an open approach to conflict resolution (e.g., disagreements are monitored, discussed openly, and resolved by the parties involved)

1□ 2□ 3□ 4 □5 □6 □7 □

3.The firm/partner is open minded and creative in its approach to problem solving (e.g., is flexible, explores its boundaries, differentiates its value proposition)

1□ 2□ 3□ 4 □5 □6 □7 □

4.The firm/partner is unpretentious and humble in its attitude to outsiders such as partners and suppliers

1□ 2□ 3□ 4 □5 □6 □7 □

5.The firm/partner is agile (e.g., quick to respond to emerging situations, reach decisions, sign agreements, enter markets, launch products, and seize opportunities)

1□ 2□ 3□ 4 □5 □6 □7 □

***Sustainable innovation behavior***

1.I am always looking for new techniques and methods.

1□ 2□ 3□ 4 □5 □6 □7 □

2.：I often come up with creative ideas.

1□ 2□ 3□ 4 □5 □6 □7 □

3.I often communicate with others and recommend my new ideas.

1□ 2□ 3□ 4 □5 □6 □7 □

4.I always try to get the resources I need to implement my new ideas.

1□ 2□ 3□ 4 □5 □6 □7 □

5.I will make proper long-term plans to implement my new ideas.

1□ 2□ 3□ 4 □5 □6 □7 □

6.On the whole, I am a sustainable innovative person.

1□ 2□ 3□ 4 □5 □6 □7 □
