# Supplementary material for: Sustainable innovation in the context of organizational cultural diversity: The role of cultural intelligence and knowledge sharing
Source: PLoS One. 2021 May 19;16(5):e0250878. doi: 10.1371/journal.pone.0250878 (PMC8133532; doi:10.1371/journal.pone.0250878)
Supplement: S2 File — (DOCX) [file pone.0250878.s003.docx]

Mean, standard deviation, maximum and minimum values of basic information and variable items in the questionnaire

| **Variable** | **Items** | **Minimum** | **Maximum** | **Mean** | **Std.** |
| --- | --- | --- | --- | --- | --- |
| **Basic Information Statistics**  （**control variable**） | Gender | 1 | 2 | 1.51 | .501 |
|  | AGE | 1 | 6 | 2.25 | 1.220 |
|  | ED | 1 | 4 | 2.99 | .682 |
|  | YEAE | 1 | 5 | 2.98 | 1.351 |
|  | GRADE | 1 | 4 | 1.59 | .890 |
|  | JOB | 1 | 8 | 5.01 | 2.747 |
|  | FIRM | 1 | 5 | 2.55 | 1.503 |
|  | IND | 1 | 8 | 5.35 | 2.428 |
|  | SIZE | 1 | 6 | 3.40 | 2.019 |
| **sustainable innovation behavior** | SI**B**1 | 1 | 7 | 5.04 | 1.297 |
|  | SI**B**2 | 1 | 7 | 4.77 | 1.297 |
|  | SI**B**3 | 1 | 7 | 4.96 | 1.304 |
|  | SI**B**4 | 1 | 7 | 5.07 | 1.301 |
|  | SI**B**5 | 1 | 7 | 5.23 | 1.245 |
|  | SI**B**6 | 1 | 7 | 4.91 | 1.194 |
| - **Cultural Intelligence** | CQ1 | 1 | 7 | 5.21 | 1.175 |
|  | CQ2 | 1 | 7 | 5.04 | 1.211 |
|  | CQ3 | 1 | 7 | 4.16 | 1.397 |
|  | CQ4 | 1 | 7 | 4.27 | 1.360 |
|  | CQ5 | 1 | 7 | 4.37 | 1.311 |
|  | CQ6 | 1 | 7 | 4.11 | 1.369 |
|  | CQ7 | 1 | 7 | 4.09 | 1.336 |
|  | CQ8 | 1 | 7 | 4.68 | 1.315 |
|  | CQ9 | 1 | 7 | 5.01 | 1.174 |
|  | CQ10 | 1 | 7 | 4.93 | 1.217 |
|  | CQ11 | 2 | 7 | 5.07 | 1.181 |
|  | CQ12 | 1 | 7 | 5.07 | 1.173 |
| **knowledge sharing** | KS1 | 1 | 7 | 4.83 | 1.291 |
|  | KS2 | 1 | 7 | 5.07 | 1.132 |
|  | KS3 | 1 | 7 | 5.14 | 1.200 |
|  | KS4 | 1 | 7 | 4.93 | 1.215 |
| **Organizational culture difference** | OCD1 | 1 | 7 | 4.24 | 1.530 |
|  | OCD2 | 1 | 7 | 4.60 | 1.427 |
|  | OCD3 | 1 | 7 | 4.55 | 1.487 |
|  | OCD4 | 1 | 7 | 4.54 | 1.403 |
|  | OCD5 | 1 | 7 | 4.21 | 1.449 |
|  | OCD6 | 1 | 7 | 4.65 | 1.391 |
|  | OCD7 | 1 | 7 | 4.53 | 1.345 |
|  | OCD8 | 1 | 7 | 4.98 | 1.302 |
|  | OCD9 | 1 | 7 | 4.77 | 1.362 |
