# Supplementary material for: Sustainable innovation in the context of organizational cultural diversity: The role of cultural intelligence and knowledge sharing
Source: PLoS One. 2021 May 19;16(5):e0250878. doi: 10.1371/journal.pone.0250878 (PMC8133532; doi:10.1371/journal.pone.0250878)
Supplement: Attachment — Submitted filename: Response to Reviewers.docx [file pone.0250878.s005.docx]

**Reply to the first reviewer**

Dear reviewer:

Thank you very much for your affirmation, which is the greatest encouragement to us!

1.Due to the limitation of Covid-19, our questionnaire survey is conducted in three ways: one is to make an appointment and mail the paper questionnaire; the other is to send and collect the electronic version of the questionnaire by E-mail; the third is to use the "Questionnaire Star" research tool. The whole questionnaire survey period was 2 months.

2.As for grass-roots employees, the research object of this paper is the individual in the enterprise, and the subject is grass-roots employees, who are the front-line employees in the workplace, accounting for 63.1%. I strongly agree with you that there is a high degree of heterogeneity between different departments. For example, the CQ and innovation of employees in R&D and sales departments should be different. However, the survey subjects in this paper do not distinguish departments, which is the research limitation of this paper. We will be paid attention to in the later research. I agree to use the industry (department) of the employee as the control variable.

Thank you very much.

With kind regards

Li jinlong Wu na Xiong shengxu

**Reply to the second reviewer:**

Dear reviewer:

Thank you very much for the affirmation of the article. We accept your opinions and will continue to improve.

1.We agree with your understanding of the term sustainable innovation behavior.To avoid confusion, we accept your suggestion and using “sustained” instead. Avoid confusion between the section you pointed out (line 113) and the explanation that follows.

2. Based on your comments, we will provide a questionnaire as supplementary information.

3. Thank you for your guidance for future research. Based on your comments, the authors of this paper plan to do further in-depth research into the differences in CQ in specific industries or in specific regions.

Thank you very much.

With kind regards

Li jinlong Wu na Xiong shengxu

**Reply to the third reviewer:**

Dear reviewer:

Thank you for your careful reading and objective evaluation,we are encouraged!

We humbly accept the hypothesis analysis problem you raised and take this problem as the research limitation of this paper. However, due to the limitation of theoretical level and language, it is difficult to revise all the assumptions. We will try our best to revise them before the publication of the manuscript. We also look forward to your further help. We will avoid this question in later writing. In addition, before the manuscript is published, we will make more efforts to make the statement more clear and try our best to do better.

Thank you very much.

With kind regards

Li jinlong Wu na Xiong shengxu
